# Google Search Trends About Systemic Psoriasis Treatment: What Do People Want to Know About Biologics and Janus Kinase Inhibitors?

**DOI:** 10.2196/62948

**Published:** 2024-10-01

**Authors:** Subin Lim, Sarah Kooper-Johnson, Courtney A Chau, Claire Chen, Fei-Shiuann Clarissa Yang, Gabriela Cobos

**Affiliations:** 1 Department of Dermatology Tufts Medical Center Boston, MA United States; 2 Icahn School of Medicine at Mount Sinai New York, NY United States

**Keywords:** Google, psoriasis vulgaris, psoriasis, systemic treatment, biologics, small molecule inhibitors, adalimumab, apremilast, methotrexate, health care delivery, skin, dermatologist, medication

## Introduction

Google is the most widely used search engine worldwide [1]. While previous studies have utilized Google machine learning algorithms to assess commonly asked questions about various medical topics [2,3], no studies have employed these tools to explore queries surrounding dermatological conditions. Recognizing the internet’s profound influence on patients, our study aimed to examine the most frequently asked questions concerning systemic treatment for psoriasis vulgaris and evaluate the quality of medical information available online.

## Methods

The Google Trends tool was utilized to compare the relative search volume (RSV) of traditional disease-modifying antirheumatic drugs, biologics, and small molecule inhibitors used to treat psoriasis between January 31, 2019, and January 31, 2024. Trade names were used. For example, “Enbrel for psoriasis” was compared with “Humira for Psoriasis.” Subsequently, the People AlsoAsked tool was utilized to generate the most asked questions about the most searched medication in each of the three categories. The questions were checked for relevance and classified based on Rothwell’s criteria (Multimedia Appendix 1). Cohen κ coefficient was calculated to determine the level of interrater agreement. Additionally, the People AlsoAsked tool was employed to extract internet sources sought by the readers. The quality of these information sources was determined based on the *The Journal of the American Medical Association* (*JAMA*) benchmark criteria [4]. Statistical analyses were performed in R 4.1.2 (R Foundation for Statistical Computing).

## Results

Adalimumab exhibited the highest search volume among all medications (RSV 1). Apremilast was the most searched among small molecule inhibitors and methotrexate among disease-modifying antirheumatic drugs (RSV 1). Adalimumab garnered the most fact-based questions overall when compared with apremilast and methotrexate (68/147, 46.3% vs 42/125, 33.6%; *P*=.04; 68/147, 46.3% vs 59/180, 32.7%; *P*=.01 for *t* tests, respectively), with the majority falling into the subcategories of technical details (36/147, 24.5%) and cost (18/147, 12.2%; Table 1).

Inquiries specifically revolved around scheduling of adalimumab administration, dietary restrictions linked to medication usage (eg, concurrent use with alcohol), and concerns about affording adalimumab.

Between apremilast and methotrexate, apremilast drew more cost-related questions (13/125, 10.4% vs 1/180, 0.5%; *P*<.001 for *t* tests), whereas methotrexate attracted more questions about its risks when compared with adalimumab (72/180, 40% vs 35/147, 23.8%; *P*=.002 for *t* tests). The interrater agreement indicated a strong agreement in question categorization (κ=0.96).

Our findings on adalimumab suggest that it has the most public awareness, possibly due in part to direct-to-patient marketing. According to data on advertisement expenses, AbbVie allocated almost US $500 million toward advertising adalimumab in 2020, while roughly half of that amount (US $202 million) was dedicated to promoting risankizumab. In the same period, Amgen invested US $150.4 million in advertising for apremilast [5].

Nonetheless, most inquiries about adalimumab centered around its cost and objective details rather than its safety. This aligns with trends observed in similar studies on rheumatoid arthritis and spinal surgeries, where individuals sought more factual information about these topics, such as the timeline for treating rheumatoid arthritis and activity restrictions related to spine surgeries [2,3]. The lower frequency of value-based questions on systemic psoriasis treatment may be due to a lack of patient understanding about the value of these medications, underscoring the need for comprehensive patient education on this topic. Alternatively, patients may be finding adequate information on these subjects from other sources, such as their dermatologists.

Furthermore, 782 websites were classified, with the majority (362/782, 46.3%) consisting of commercial sites such as Healthline. Social media websites accounted for 24.4% (191/782), government-based websites such as PubMed accounted for 15.2% (119/782), academic websites for 12.6% (98/782), and medical practice websites for 1.5% (12/782). In assessing the quality of these sources, commercial and government websites scored the highest average based on the *JAMA* benchmark criteria, with 3.1 and 3.2 points out of 4, respectively. Medical practice websites scored the lowest, with an average of 1.0 points (Table 2).

**Table 1:** Relative proportion of question type for apremilast, adalimumab, and methotrexate and significance of difference.

Question category		Apremilast (n=125), n (%)	Adalimumab (n=147), n (%)	MTX^a^ (n=180), n (%)	Apremilast vs adalimumab, *P* value (*t* test)	Apremilast vs MTX, *P* value (*t* test)	Adalimumab vs MTX, *P* value (*t* test)
**Fact**		42 (33.6)	68 (46.3)	59 (32.7)	.04^b^	.88	.01^b^
	Cost	13 (10.4)	18 (12.2)	1 (0.5)	.64	<.001^b^	<.001^b^
	Mechanism	6 (2.4)	6 (4.1)	6 (3.3)	.44	.64	.72
	Technical	15 (12.0)	36 (24.5)	44 (24.4)	.01^b^	.007^b^	.98
	Timeline of treatment	8 (6.4)	8 (5.4)	8 (4.4)	.73	.45	.68
**Policy**		44 (35.2)	37 (25.2)	75 (41.7)	.07	.26	.002^b^
	Risks	42 (33.6)	35 (23.8)	72 (40.0)	.07	.26	.002^b^
	Indications	2 (1.6)	2 (1.4)	3 (1.7)	.84	.96	.86
**Value**		39 (31.2)	46 (29.2)	46 (25.5)	.72	.28	.45
	Evaluation	22 (17.6)	31 (17.7)	31 (17.2)	.98	.93	.91
	Prognosis	6 (4.8)	7 (4.8)	8 (4.4)	.97	.88	.89
	Timeline of clinical course	11 (8.8)	9 (6.1)	7 (3.8)	.40	.07	.35

^a^MTX: methotrexate.

^b^Statistical significance.

**Table 2:** Evaluation of internet source categories and quality according to *The Journal of the American Medical Association* (*JAMA*) benchmark criteria.

Category	Description	Internet sources (n=782), n (%)	Average source quality score (out of 4)
Commercial	Commercial organization that positions itself as a source of health information, includes medical device and pharmaceutical companies (eg, Healthline, WebMD)	362 (46.3)	3.1
Academic	Institution with a clear academic mandate, including universities, academic medical centers, academic societies, and journals (eg, Mayo Clinic)	98 (12.5)	2.3
Government	Websites ending in .gov or maintained by a national government (eg, PubMed)	119 (15.2)	3.2
Medical practice	Local hospital or dermatology practices without an academic affiliation	12 (1.5)	1
Social media	Websites maintained by nonmedical organizations primarily designed for information sharing between internet users, including health blogs, internet forums, and support groups	191 (24.4)	2.1

## Discussion

Our analyses indicate that users are being directed to commercial and government-based websites most often when seeking information about psoriasis treatment. This is reassuring, as these websites received the highest scores based on source quality criteria. Surprisingly, academic websites scored lower on average, similar to social media websites. Finally, although individual medical practice websites were not referred to as often, they scored only 1 out of 4 on average, indicating an area for improvement both for these practices and the search engine. Enhancing the visibility and content quality of medical practice websites as determined by the *JAMA* benchmark criteria and optimizing search engine algorithms to prioritize higher-quality sources could improve patient access to reliable health information.

In summary, given that the internet has a substantial impact on the dissemination and understanding of health-related information, dermatologists should consider tailoring their discussions when counseling patients on systemic medications for psoriasis. Emphasis should be placed on addressing a medication’s administration schedule, dietary restrictions associated with its use, cost considerations, and side effect profile relative to alternative options. Additionally, dermatologists can guide patients on how to identify and access high-quality online resources, empowering them to make more informed decisions about their health. Study limitations include potential question comprehensiveness and the evolving nature of medication concerns over time.

## Appendix

Multimedia Appendix 1 Question classification based on Rothwell's criteria and subcategories specific to the dataset.

## Abbreviations

*JAMA*: *The Journal of the American Medical Association*
RSV: relative search volume
