# Supplementary figures and images for: Google Search Trends About Systemic Psoriasis Treatment: What Do People Want to Know About Biologics and Janus Kinase Inhibitors?
Source: JMIR Dermatol. 2024 Oct 1;7:e62948. doi: 10.2196/62948 (PMC11480731; doi:10.2196/62948)

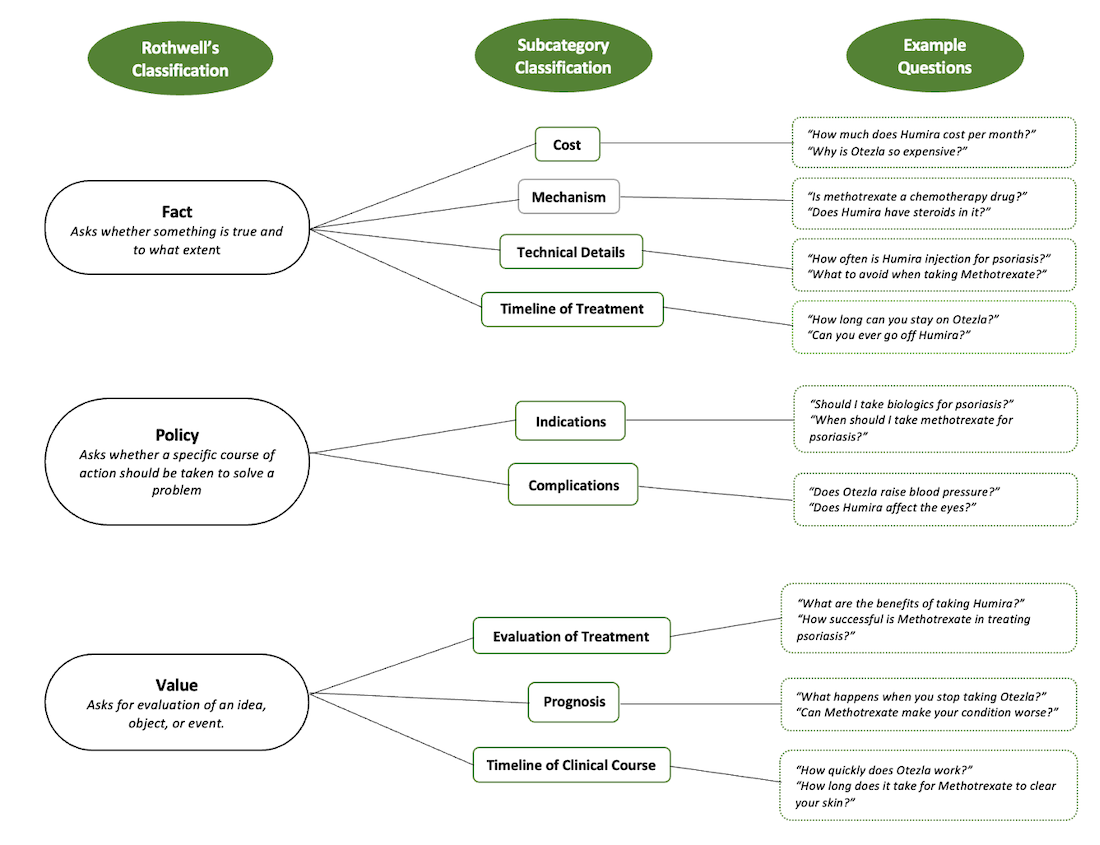

Supplement: Multimedia Appendix 1 [file derma_v7i1e62948_app1.png]
